# Theta and high-beta networks for feedback processing: a simultaneous EEG–fMRI study in healthy male subjects

**DOI:** 10.1038/tp.2016.287

**Published:** 2017-01-31

**Authors:** C Andreou, H Frielinghaus, J Rauh, M Mußmann, S Vauth, P Braun, G Leicht, C Mulert

**Affiliations:** 1 Psychiatry Neuroimaging Branch, Department of Psychiatry and Psychotherapy, University Medical Center Hamburg-Eppendorf, Hamburg, Germany; 2 Center for Gender Research and Early Detection, University of Basel Psychiatric Clinics, Basel, Switzerland

## Abstract

The reward system is important in assessing outcomes to guide behavior. To achieve these purposes, its core components interact with several brain areas involved in cognitive and emotional processing. A key mechanism suggested to subserve these interactions is oscillatory activity, with a prominent role of theta and high-beta oscillations. The present study used single-trial coupling of simultaneously recorded electroencephalography and functional magnetic resonance imaging data to investigate networks associated with oscillatory responses to feedback during a two-choice gambling task in healthy male participants (*n*=19). Differential associations of theta and high-beta oscillations with non-overlapping brain networks were observed: Increase of high-beta power in response to positive feedback was associated with activations in a largely subcortical network encompassing core areas of the reward network. In contrast, theta-band power increase upon loss was associated with activations in a frontoparietal network that included the anterior cingulate cortex. Trait impulsivity correlated significantly with activations in areas of the theta-associated network. Our results suggest that positive and negative feedback is processed by separate brain networks associated with different cognitive functions. Communication within these networks is mediated by oscillations of different frequency, possibly reflecting different modes of dopaminergic signaling.

## Introduction

*'*Once bitten, twice shy': adaptive behavior depends on the ability to recognize contingencies and to use them to make predictions about future events. These functions are carried out by the reward system, core components of which include the ventral tegmental area and substantia nigra, ventral and dorsal striatum, and dorso-/ventromedial prefrontal areas.^1, 2^ Research into the reward system is relevant for our understanding of psychiatric disorders such as psychotic, mood and substance disorders.

The reward system does not act in isolation; its output needs to be evaluated and also registered in memory. To achieve these purposes, the aforementioned core reward regions interact with several other areas—most notably regions involved in cognitive and emotional processing such as the medial and lateral prefrontal cortex, hippocampus and amygdala.^1^ These various components need to be flexibly and differentially recruited depending on the specific context (for example, significance for survival, conflicts between short- and long-term rewards and so on). One key mechanism through which this is achieved is oscillatory activity: neuronal oscillations enable communication between distant brain areas, with oscillations of different frequency corresponding to different network configurations.^3, 4, 5^ Therefore, neuronal oscillations of varying frequency are a plausible candidate as the mechanism of flexible communication within the reward system.

Several electroencephalography (EEG) studies have provided evidence for frequency-specific responses to different reward-related stimuli. In gambling paradigms, processing of positive outcomes is mainly associated with oscillations in the high-beta/low-gamma frequency range. On the other hand, losses are accompanied by an increase in the power and synchronization of oscillations in the theta frequency range^6, 7, 8^ and, partly associated with these, by a negative event-related potential with a midfrontal scalp distribution, the feedback-related negativity.^9^ Beta- and theta-band oscillations respond to different features of the feedback stimulus: For example, the theta-band response has been reported to be mainly driven by feedback valence,^8, 9^ whereas high-beta oscillations are affected by additional aspects of reward-related stimuli such as their probability and magnitude.^6, 8, 10^ Moreover, previous studies by our group and others indicate that the two types of oscillatory response are differentially associated with trait impulsivity: Both in healthy subjects^11, 12, 13^ and in patients with borderline personality disorder and alcohol dependence,^14, 15^ impulsivity is associated with dampened theta-band oscillatory responses to negative feedback, an effect that involves the dorsal anterior cingulate cortex (dACC) and possibly also lateral prefrontal areas;^14^ in contrast, beta oscillatory responses to reward are not correlated with trait impulsivity.^13, 14^

The above dissociation supports the notion of a frequency-specific, context-dependent modulation of the reward system. However, it is still unclear whether the latter involves separate sub-networks within the reward system, or rather represents the same components interacting with each other by means of different oscillatory processes. On the basis of theoretical considerations, it was proposed that the former is the case, with high-beta activity originating in ventromedial, and theta activity in dorsomedial, prefrontal areas.^16, 17^ However, EEG studies have failed to conclusively confirm or disconfirm this hypothesis; the two types of oscillatory response have a largely overlapping midfrontal topography, and source localization studies have provided partly inconsistent results. Theta-band oscillations and the closely associated feedback-related negativity in response to negative feedback have often been reported to originate in the anterior cingulate cortex (ACC),^16, 18^ but other generators such as the posterior cingulate cortex^19^ or basal ganglia^20, 21^ have also been proposed. High-beta responses to reward, on the other hand, have been localized in dorsolateral prefrontal areas^14, 22^ but, in one case, also in the ACC.^14^

The above discordant findings exemplify limitations of EEG-based approaches, resulting from the lack of a unique solution to the inverse problem of cortical source localizations based on scalp-recorded activity. Another limitation of EEG is that it is restricted in its capacity to detect activity in deep-located structures of the brain; this constrains its usefulness when investigating the reward system, which comprises several subcortical components. These limitations can be overcome with use of multimodal imaging techniques that combine EEG and functional magnetic resonance imaging (fMRI) analyses, profiting both from the superiority of EEG in assessing the temporal characteristics of neural oscillations and from the excellent spatial resolution of fMRI.^23, 24, 25^

So far, only two studies have used multimodal techniques to depict networks associated with the feedback-related negativity^26^ or high-beta oscillatory responses to reward.^27^ Their findings suggest that the two EEG measures correspond to different networks: the feedback-related negativity in response to negative feedback was associated with activations in a purely cortical network,^26^ whereas the high-beta oscillatory responses to positive feedback corresponded to a network comprising not only lateral frontal, but also striatal and hippocampal areas.^27^ However, it should be kept in mind that comparability of the above two studies is limited due to the different paradigms used. According to recent evidence,^28^ the same brain areas may communicate in different frequencies depending on the exact cognitive operations involved, even within the same cognitive domain (that is, learning from feedback).

Prompted by the above, the aim of the present study was to investigate the networks associated with oscillatory responses to feedback within the context of the same gambling paradigm, using EEG-informed fMRI. Based on existing literature, we hypothesized that theta-band oscillations upon loss would be associated with activations in a frontoparietal network including the ACC, whereas high-beta oscillations in response to positive feedback would involve activations of frontal, striatal and hippocampal areas. Furthermore, we expected that processing of different feedback dimensions (valence vs magnitude) would implicate different brain areas in both frequency ranges; the latter assumption was not specified further owing to the scarcity of related previous findings. A secondary aim of the study was to explore the association between trait impulsivity and theta-associated activations in response to negative feedback. According to our previous results, we expected a negative correlation between impulsivity scores and theta-band-associated activations in the dACC and/or lateral prefrontal areas.

## Materials and methods

### Participants

The study was conducted in accordance with the Declaration of Helsinki and was approved by the ethics committee of the Medical Council of Hamburg. All the participants provided written informed consent.

Twenty-two healthy male individuals (age 23.67±3.2 years) were recruited among students of the University of Hamburg. The sample size was defined based on previous studies by our group on feedback processing.^13, 14^ All the participants were nonsmokers and had normal or corrected-to-normal vision. Exclusion criteria were lifetime psychotic, bipolar or substance-use disorders, depressive or anxiety disorders in the past year, neurological or major somatic illnesses, and psychotropic or any other medication known to affect cognitive functions. Three subjects were excluded from the analyses (one because he later admitted to daily cannabis consumption in the week preceding the testing session, one because of major head movement and one because of very poor EEG data quality). Thus, 19 participants were included in the final analysis.

In all the participants, trait impulsivity was assessed with the Barratt Impulsiveness Scale (BIS), a 30-item Likert-type self-report questionnaire yielding scores for attentional, motor and non-planning impulsivity.^29^ The BIS has been widely used in similar studies and has good reliability and validity.^30^ Moreover, a general screening of personality attributes was carried out with the German version of the NEO Five-Factor Inventory,^31^ a self-rating instrument containing 60 items that are rated on a five-point Likert-type scale across five personality dimensions: neuroticism, extraversion, openness to experience, conscientiousness and agreeableness.

### Gambling task

The participants performed a computerized two-choice gambling task (adapted from Gehring and Willoughby^32^) that has been used in previous EEG studies by our group and others.^8, 13, 14, 33^

Presentation version 17, installed on a computer set in a monitoring room shielded from the MR scanner, was used for stimulus presentation. The experiment consisted of four blocks of 100 trials each. At the beginning of each trial, two numbers (25 and 5) were presented on the screen in randomized position order ([25] [5] or [5] [25]). The participants were instructed to choose one of the two numbers by button press within 1 s of the stimulus onset. Two seconds after trial onset, the selected number was set to bold; if the participant had failed to press a button in the required time, the trial was dismissed. After a further delay of 2 s, one of the numbers randomly turned green and the other red, indicating whether the selected amount (25 or 5) was added (green—win feedback) or subtracted (red—loss feedback) from the participant's account. The trial ended with a 2 s display presenting the current account balance. A 2 s fixation square preceded the next trial.

The participants were instructed in a standardized manner and practiced the paradigm in advance. They were informed that their aim was to gain as many points as possible, that loss and gain events occurred at equal probability and that in each trial they were free to choose the high- or low-risk option without any constraints. The participants were reimbursed with 30€ for study participation.

### EEG acquisition

EEG was recorded during fMRI acquisition using BrainVision Recorder (Version 1.10, Brain Products, Munich, Germany) and MR-compatible AC-amplifiers (BrainAmp MRplus; Brain Products). The electrode cap (BrainCapMR 64, Brain Products) contained 62 active sintered silver/silver chloride EEG electrodes positioned according to a modified 10/10 system; FCz served as reference and AFz as ground; an EOG electrode under the left eye and an ECG electrode recorded eye movement and data for cardioballistogram correction, respectively. The ribbon cable connecting the electrode wires and amplifiers was fixated with sand bags on foam cushions to avoid artifacts generated by the scanner's vibrations. Electrode skin impedance was kept below 10 kΩ. The data were collected with a sampling rate of 5000 Hz and an amplitude resolution of 0.5 μV.

### fMRI acquisition

Imaging was performed on a 3-Tesla MR scanner (Magnetom Trio, Siemens, Munich, Germany) equipped with a 12-channel head coil. Twenty-five slices were recorded using a standard gradient echo-planar imaging (EPI) T2*-sensitive sequence for functional blood-oxygen-level-dependent (BOLD) imaging. For each block, there were 530 volumes (TR=2 s; TE=25 ms; FOV=216 mm; matrix=108 × 108; continuous slice acquisition; slice thickness=3 mm; interslice gap=1 mm). The vacuum pump of the MRI scanner was switched off during acquisition, to avoid EEG artifacts in the high-frequency ranges. A high-resolution (voxel size 1 × 1 × 1 mm) T1-weighted anatomical image (MPRAGE) was acquired for each subject in the same position as the EPI images.

### EEG preprocessing and time-frequency analysis

Brain Vision Analyzer Version 2.0 (Brain Products) was used for offline EEG data preprocessing and analysis. The continuous MR-Artifact was corrected by generating a sliding average template using baseline correction. The data set was resampled to a sampling rate of 500 Hz and filtered with a 50 Hz low-pass (slope 12 dB/oct) and 0.1 Hz (slope 48 dB/oct) high-pass Butterworth zero-phase filter. For cardioballistic artifact correction, a pulse template was semi-automatically detected and marked in the electrocardiogram channel, then used to subtract the cardioballistic artifact from recordings. Prominent non-stereotyped artifacts such as movement artifacts and channel drifts were removed by visual inspection. Independent component analysis (restricted biased Infomax algorithm) was applied to eliminate further artifacts; components indicating blinks and eye-movements, residual gradient and head movement artifacts were detected and removed on the basis of their power spectrum and topography. Subsequently, the EEG signal was re-referenced to a common average reference and segmented into periods of 3 s, starting 1800 ms before the feedback stimulus. Baseline correction for the 200 ms pre-stimulus interval was applied. An automatic artifact correction procedure rejected segments that contained voltage steps higher than 50 μV, amplitudes exceeding ±95 μV or a difference higher than 200 μV between the highest and lowest value, or activity below 0.5 μV.

Time-frequency information was extracted at the single-trial level for EEG activity at electrode Fz (similar to previous studies by our group^13, 14^) using wavelet convolution for the frequencies from 2 to 50 Hz (complex Morlet wavelet, 25 frequency steps distributed on a logarithmic scale, Morlet parameter c=6, Gabor Normalization). Oscillatory power at each time point and frequency layer was divided by a baseline norm value *n* representing the sum of values across the 200 ms pre-stimulus baseline, weighted by the relative length of the baseline interval with respect to total segment length. In this way, power changes with respect to the pre-stimulus baseline were assessed, rather than absolute power. Layers with central frequencies of 5.1Hz (range: 4.4–5.8 Hz) and 25.5 Hz (range: 22–29 Hz) were extracted to investigate theta and high-beta activity, respectively. Markers indicating the maximum peak power 100–600 ms post-stimulus for theta and 100–500 ms post-stimulus for high-beta were set.

The effects of feedback valence (gain vs loss) and magnitude (25 vs 5 points) on theta and high-beta power were assessed with separate linear-mixed models (LMMs), which were preferred over repeated-measures ANOVAs because they are better suited to address inter-subject variability. Dependent variables for the two linear-mixed models were averaged peak theta and high-beta values over trials; valence and magnitude were repeated-measures fixed-effect factors. The valence × magnitude interaction was initially included in the models but subsequently removed, as it was not significant in either of the two analyses (both *P*>0.180). Both linear-mixed models used the maximum-likelihood estimation algorithm and a diagonal covariance structure; subject ID was included as a random factor.

### fMRI preprocessing and analysis

The fMRI data were processed using standard procedures implemented in the Statistical Parameter Mapping software (SPM12, Wellcome Department of Imaging Neuroscience, London, UK, www.fil.ion.ucl.ac.uk/spm/). The first five volumes of each block were discarded to allow for MRI saturation effects. The preprocessing included slice timing, realignment, registration to standard space (Montreal Neurological Institute) and spatial smoothing with an 8 mm Gaussian kernel.

BOLD responses to feedback stimuli were examined using the general linear model approach. For first-level analyses, the following conditions were modeled as regressors through convolution with a canonical hemodynamic response function: (a) the four conditions of feedback (large gain, large loss, small gain, small loss); (b) initial stimulus presentation; (c) motor response; (d) anticipation phase; and (e) presentation of the account balance. Motion parameters (*n*=6) were included in the model as regressors of no interest.

### EEG-informed fMRI analysis

Coupling effects of theta and high-beta power with BOLD activity were investigated in two separate general linear models. For each condition (large gain, large loss, small gain, small loss), a parametric modulator corresponding to single-trial (theta or high-beta) oscillatory power measured at Fz was added to the respective regressor representing onsets of the events of interest in the design matrix. To remove shared variance between regressors and parametric modulators, the latter were orthogonalized with respect to the former by subtracting the mean (theta or high-beta) oscillatory power within each block and condition from the single-trial power for the corresponding condition.

First-level contrasts were calculated for each feedback condition compared with baseline, and entered into a second-level flexible factorial model with three factors (valence, magnitude and subject ID as a random effect). Analyses were carried out for valence (gain > loss, loss > gain) and magnitude contrasts (large > small, small > large feedback). Effects observed at *P*<0.001 and surviving a false discovery rate (FDR) correction at the cluster level at *P*(FDR)<0.05 are reported as significant for fMRI analyses. EEG–BOLD coupling analyses typically produce weak effect sizes because of the low signal-to-noise ratio in single EEG trials. Therefore, we used a more lenient threshold of *P*<0.005 (uncorrected) with a cluster extent of 100 voxels.

### Correlations with trait impulsivity

We used a functional region-of-interest approach to investigate correlations between BIS subscales and theta-associated activations upon loss feedback: Using MarsBar (marsbar.sourceforge.net), spherical regions of interest with a 5 mm radius were built around the peak voxel of each cluster that achieved significance for the loss vs gain contrast in the theta-band EEG–BOLD coupling analysis. The mean of the linear fit coefficient of all voxels within the sphere was used as the region-of-interest summary measure for correlations. As there were no significant deviations from normality (Wilcoxon's test), Pearson's *r* was used for correlational analyses. The Benjamini and Hochberg FDR method^34^ was used to correct for multiple testing. Although our hypothesis was specific to the theta band and the loss vs gain contrast, for comparison we also conducted similar correlational analyses for the high-beta band and the opposite (that is, gain vs loss) contrast.

## Results

The following personality dimension scores (means±s.d.) were derived from the NEO Five-Factor Inventory: Neuroticism 12.58±6.3; extraversion 28.89±6.1; openness to experience 36.89±6.39; agreeableness 34.44±8.7; conscientiousness 36.53±5.5.

In six participants, only three blocks were available for analysis because of technical problems during acquisition (*n*=2), significant artifacts in single blocks that could not be removed with the procedures described above (*n*=2), and selection of the same number (25 or 5) throughout the block, resulting in null regressors in the general linear model analysis (*n*=2). For these participants, first-level general linear models were constructed on the basis of the three remaining blocks, and first-level contrasts were adjusted for the number of blocks in all the participants. All the results reported below are based on the same number of blocks in all participants. Only significant results are reported.

### EEG analyses

**Figure 1:**
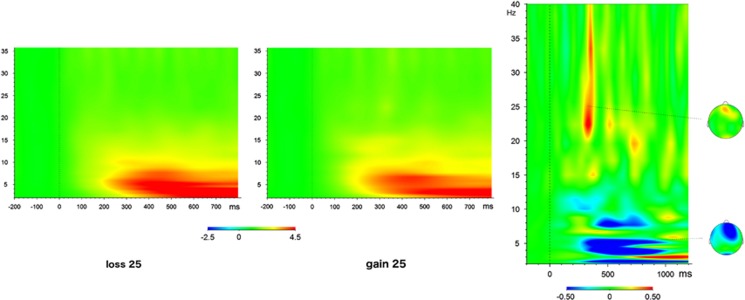
Time-frequency plot and scalp topographies for theta (5.1 Hz) and high-beta (25.5 Hz) oscillatory responses to gain vs loss feedback (time point 0) in the high-magnitude condition (right) and in each condition separately (left). Normed values with respect to a 200 ms pre-stimulus baseline are depicted.

The main effect of valence was significant both in the theta (F(1,38.00)=6.913, *P*=0.012) and high-beta band (F(1,54.87)=7.729, *P*=0.007). The direction of effects was as expected: power was increased upon negative feedback in the theta band, and upon positive feedback in the high-beta frequency band (Figure 1). There were no significant magnitude effects in either frequency band (theta F(1,42.51)=1.348, *P*=0.252; beta F(1,53.96)=0.25, *P*=0.619).

### fMRI analyses

**Figure 2:**
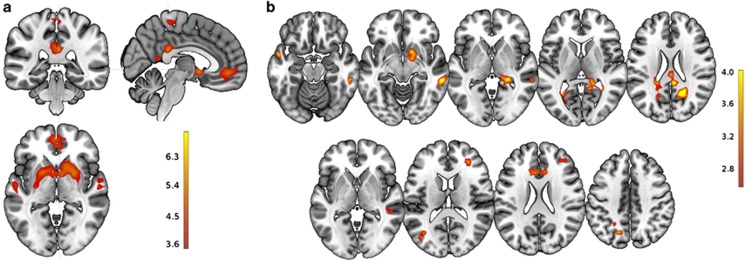
(**a**) Areas showing greater BOLD response for gain vs loss feedback (single-voxel *P*<0.001, *P*(FDR)<0.05 at the cluster level). (**b**) EEG–fMRI fusion analysis results (single-voxel *P*<0.005, *k*=100): Areas showing high-beta-band-associated activations for the contrast gain > loss feedback (top row) and theta-band-associated activations for the contrast loss > gain (bottom row). The opposite contrasts did not yield significant results in any of the above cases. BOLD, blood-oxygen-level dependent; EEG, electroencephalography; FDR, false discovery rate; fMRI, functional magnetic resonance imaging.

For the gain > loss contrast, significant activations were observed in two large clusters that included the ventral striatum, putamen, caudate nucleus, amygdala and hippocampi bilaterally, but also in anterior and posterior medial areas, and bilateral lateral temporal areas (Figure 2a and Table 1).

The magnitude contrast (large > small) revealed significant activations in the dACC, posterior medial, and occipital lateral areas (Table 1). Finally, the small > large contrast revealed activations in the right temporoparietal junction and the left lateral prefrontal cortex (Table 1).

### Oscillatory power coupling with BOLD activity

#### High-beta frequency band

Regions that showed increased BOLD activity for the contrast gain > loss included the right ventral striatum (including the nucleus accumbens) and amygdala, medial posterior and parahippocampal areas bilaterally, and lateral temporal areas bilaterally (Figure 2b and Table 2); activations in posterior areas and the right lateral temporal cortex achieved significance at a cluster-corrected threshold of *P*(FDR)<0.05.

The magnitude contrast (large > small) revealed high-beta-associated activity in the ACC (Table 2).

#### Theta frequency band

For the loss > gain contrast, significant associations with theta power were observed in the dACC, right dorsolateral prefrontal cortex (DLPFC), left and right temporoparietal junction, and left superior parietal cortex (Figure 2b and Table 3).

The magnitude contrast (large > small) yielded significant results in right inferomedial temporal areas (Table 3).

#### Correlations of theta- and high-beta-associated BOLD activations with impulsivity

Significant negative correlations with BIS subscales were observed for theta-associated activity upon loss in the following areas: (a) right DLPFC with BIS non-planning score (*r*=0.641, *P*=0.003) and BIS attention (*r*=0.551, *P*=0.014); (b) left superior parietal cortex with BIS non-planning score (*r*=0.531, *P*=0.019). Trend-wise correlations were also observed for the dACC region of interest with BIS attention (*r*=0.428, *P*=0.068) and BIS motor impulsivity (*r*=0.439, *P*=0.060). After correction for multiple testing, the correlation of right DLPFC theta-associated activity with BIS non-planning score remained significant (*P*=0.046), while the correlations of right DLPFC with BIS attention and left superior parietal cortex with BIS non-planning only achieved a trend level (both *P*=0.097).

In the high-beta frequency band, there was a significant negative correlation between activity in the right middle temporal cortex and BIS attention (*r*=−541, *P*=0.015), which disappeared after correction for multiple testing (*P*=0.369).

## Discussion

The present study used a gambling paradigm and single-trial coupling of simultaneously recorded EEG and fMRI data to investigate brain areas associated with theta and high-beta oscillatory responses to feedback. High-beta oscillations in response to gain and theta oscillations in response to loss stimuli were associated with activations in non-overlapping brain areas. Moreover, there was evidence that trait impulsivity correlated negatively with theta-associated activity upon negative feedback in some components of the respective network.

The network associated with high-beta oscillatory responses to gain involved regions typically associated with reward (ventral striatum) and memory processing (hippocampus, anterior lateral temporal cortex). It also included the posterior cingulate cortex; although this region is typically associated with the default mode network,^35^ it has been consistently implicated in reward processing, and especially positive outcome processing, by meta-analyses of fMRI data.^36, 37^ Its exact role is unclear, but studies in non-human primates suggested that it may mediate the integration of stimulus characteristics to motivate a shift in behavior.^38^ Thus, our findings are consistent with the proposition^17^ that oscillations in the high-beta/low-gamma frequency range may mediate the synchronization of brain regions involved in learning from positive feedback. A similar high-beta-/low-gamma-associated network was reported by a previous study by Mas-Herrero *et al.*,^27^ in which a different multimodal imaging technique was used to assess a gambling paradigm. A notable difference is the absence of beta-associated prefrontal cortex activations in our study.^27^ This might be attributed to subtle differences in the gambling paradigms used: The study by Mas-Herrero *et al.* included a trial-by-trial manipulation of the probability of winning (25, 50 or 75%), a dimension known to affect high-beta/low-gamma oscillations in response to reward.^10^ Moreover, their paradigm included different winning probabilities for different stimuli and thus conceivably promoted the use of explicit strategies to optimize gains more than the paradigm we implemented, in which the probability of winning was determined entirely by chance. Interestingly, using the same paradigm in a previous EEG study,^14^ we observed frontal cortex activations when contrasting only the two maximum feedback conditions (gain 25 vs loss 25), which are also those with the greatest influence on behavior.^32^ Thus, it may be that frontal activations are dependent on the usefulness of feedback for behavioral adjustments; this is consistent with the results of a previous EEG source localization study,^22^ in which high-beta-associated DLPFC activity was only observed when stimulus-reward contingencies could be used to optimize performance (see also below, section on dACC and feedback magnitude).

The theta-band response to loss events was associated with a different network comprising mainly frontoparietal areas. All these areas have been associated with negative feedback in two previous studies that used different EEG-derived components to inform fMRI analyses.^26, 39^ Moreover, an EEG study implicated theta-band synchronization of midfrontal with dorsolateral prefrontal and parietal areas in feedback processing.^40^ The parietal cortex has been associated with attentional processes, and the DLPFC with cognitive monitoring and control; both of these areas have been associated with strategy switching in learning tasks.^41, 42^ Therefore, their theta-mediated synchronization with the dACC conceivably reflects the mobilization of attentional and cognitive resources in the face of a negative outcome necessitating a new strategy. In line with this view, it has been suggested that theta oscillations have a role in processing feedback stimuli that indicate a need for a behavioral change.^28^

The above dissociation between activations associated with high-beta and theta oscillations is consistent with our hypotheses and existing theoretical accounts of feedback-based learning.^16^ Moreover, it entails the possibility that the two networks are differentially linked to the dopamine system. Midbrain dopamine neurons exhibit two modes of signaling patterns *in vivo*: low-frequency (<10 Hz) discharges in a pacemaker-like fashion, and transient high-frequency activity (15–30 Hz).^43^ It is assumed that low-frequency activity regulates tonic levels of dopamine,^44, 45^ while high-frequency activity gives rise to phasic dopamine responses.^45, 46^ Converging evidence suggests that these two types of dopaminergic activity have quite different functions. Phasic dopamine signals encode prediction error signals and are essential for processing of positive feedback in the ventral striatum, possibly reinforcing behaviors that lead to reward by regulating synaptic plasticity.^47^ On the other hand, tonic dopamine levels in the prefrontal cortex have been suggested to be relevant for motivation,^48, 49^ as well as for producing a sustained activation state that promotes attentional and monitoring functions while the individual is pursuing a goal.^44, 48, 50^ These two different modes of dopamine signaling might be reflected in the two different activation patterns we observed—a high-frequency network in reward areas and a low-frequency network in areas associated with cognitive monitoring and control. However, it remains to be determined how the slow dynamics of the mesocortical, low-frequency dopamine system might trigger the fast theta response, occurring within milliseconds from negative feedback (see for example, Jocham and Ullsperger^51^).

The dACC was prominently involved in the processing of negative feedback, in line with the proposed role of this region in using action outcomes to guide future behavior.^52, 53^ However, there was also an association with feedback magnitude. This finding might reflect a conflict monitoring function of the dACC (see for example, Knutson *et al.*^54^), as larger gains in our paradigm also entailed the possibility of higher risk. Alternatively, it may relate to the significance of feedback magnitude for behavioral adaptation:^32^ In a monkey study, reward-associated beta oscillations in the ACC were dependent on the usefulness of feedback in terms of learning.^55^ Notably, the two aspects of dACC involvement—processing of feedback valence vs magnitude—were mediated by oscillations of different frequency, and were localized in adjacent, but distinct areas of the dACC. These results are in line with the view of the dACC as a region with significant heterogeneity, suggested to consist of sub-networks that deal with different feedback dimensions.^56^ They are also consistent, to revisit the points made above, with the assumption that the dACC subserves different aspects of decision-making by responding differently to changes in tonic vs phasic dopamine firing rate.^48^ It is argued that tonic dopamine levels enable the 'online' maintenance of task-related information through D1 receptor modulation, whereas phasic dopamine changes mediate information updating and outcome appraisal by acting on D2 receptors^44, 48, 57^—although there are alternative accounts.^58^ In this framework, increased theta-band activity in the dACC following negative outcomes could, as detailed above, reflect reallocation of cognitive resources in the face of negative feedback, whereas high-beta activity in response to large wins or losses might serve to flag events that are important for the ongoing cost-benefit analysis of selection behavior.

Various aspects of trait impulsivity correlated negatively with theta-associated BOLD activity in the right DLPFC and, to a less-pronounced degree, with the left superior parietal cortex. This finding expands upon previous studies associating trait impulsivity with a deficit in theta oscillatory responses to negative feedback.^12, 13, 14, 15^ It has been suggested that this deficit represents a 'reward deficiency syndrome', whereby a reward system dysfunction leads to stimulation-seeking behaviors such as drug abuse and impulsivity.^59^ Our results only partially confirm this hypothesis: according to the foregoing considerations, reduced theta responses to negative feedback are more likely to represent a deficit in cognitive processes that use reward system output to guide behavior, rather than dysfunctional reward mechanisms *per se*. However, this conclusion needs to be confirmed with studies in clinical populations, as the present study only investigated normal impulsivity variations in healthy individuals.

The present study included only male participants in an effort to increase sample homogeneity, given reports of gender differences in negative feedback processing.^12, 60^ However, this might entail limitations for the generalizability of results, which remain to be confirmed. A further limitation is that the simple gambling paradigm we used did not allow monitoring of participants' expectations, thus making it impossible to disentangle the effects of reward and loss from those of prediction error. This should be kept in mind when considering the above interpretation of results and the postulated role of the dACC, given that midfrontal theta-band oscillations have been implicated in the processing not only of negative feedback, but also of unsigned (that is, valence-independent) prediction error.^26, 61^ Finally, although theta and high-beta are the most intensively studied frequencies in the context of reward and loss, oscillations in other frequency ranges such as alpha and delta have been also reported to be relevant for feedback processing.^13, 62, 63^ A detailed assessment of these frequencies would well exceed the scope of the present study, but might be an interesting goal for further studies.

In summary, we were able to show that positive and negative feedback is processed by separate brain networks associated with different cognitive functions, and possibly with different aspects of dopaminergic signaling. Communication within each of these two networks, but also processing of different feedback dimensions within the same region (dACC), were mediated by oscillations of different frequency, speaking for a prominent role of frequency-specific neuronal oscillations in the flexible, context-dependent adaptation of reward-related areas. Trait impulsivity was associated with decreased theta-associated activation in frontoparietal areas, suggesting a deficit in attentional and monitoring processes associated with reward processing in impulsive subjects.

## Figures and Tables

**Table 1:** fMRI activations

*Anatomical area*	*Coordinates*	P*(FDR)*	*Size*	z*-score*
*Gain > loss*				
L putamen	−14 2 −12	<0.001	5064	7.27
R amygdala	14 4 −12			6.60
L putamen	−30 −4 6			4.74
				
R posterior cingulate	2 −34 26	<0.001	1141	4.78
L precuneus	−8 −64 34			4.55
L precuneus	−6 −48 14			4.52
				
L precentral gyrus	−32 −28 56	0.001	346	4.47
L postcentral gyrus	−40 −20 52			3.94
L postcentral gyrus	−34 −24 48			3.85
				
R postcentral gyrus	44 −16 36	0.006	242	4.38
R posterior cingulate	28 −16 50			3.87
R precentral gyrus	36 −16 44			3.84
				
L superior frontal gyrus	−14 36 48	0.004	271	4.32
L superior frontal gyrus	−14 48 36			4.16
				
L superior temporal gyrus	−56 −10 0	0.001	400	4.12
L superior temporal gyrus	−52 −32 14			3.76
L middle temporal gyrus	−54 −18 0			3.69
				
R paracentral lobule	2 −32 64	<0.001	472	3.95
R middle cingulate	6 −20 46			3.82
L paracentral lobule	−6 −34 68			3.74
				
R superior temporal gyrus	58 −14 −2	0.001	379	3.92
R superior temporal gyrus	66 −16 10			3.68
R superior temporal gyrus	62 −4 0			3.62
				
*25>5-Point feedback*				
L superior occipital gyrus	−16 −96 10	<0.001	550	6.00
L lingual gyrus	−14 −86 −8			4.63
L fusiform gyrus	−24 −78 −8			4.29
				
R cuneus	20 −92 14	<0.001	833	5.63
R lingual gyrus	16 −80 −8			5.42
R superior occipital gyrus	24 −88 22			4.40
				
R ACC	4 28 24	0.008	238	4.03
L ACC	−4 20 16			3.98
L ACC	0 36 20			3.86
				
*5>25-Point feedback*				
L middle frontal gyrus	−40 18 50	0.014	238	4.77
L middle frontal gyrus	−42 22 38			3.51
				
R angular gyrus	58 −54 32	<0.001	1050	4.54
R superior temporal gyrus	56 −50 22			4.20
R middle temporal gyrus	42 −52 20			4.18

Abbreviations: ACC, anterior cingulate cortex; FDR, false discovery rate; fMRI, functional magnetic resonance imaging; L, left; R, right.

**Table 2:** EEG–fMRI coupling—high-beta-associated activations

*Anatomical area*	*Coordinates*	P*(FDR)*	*Size*	z*-score*
*Gain > loss*				
R middle temporal gyrus	60 −38 −4	0.042	306	4.40
R middle temporal gyrus	58 −36 −14			3.76
R inferior temporal gyrus	48 −24 −24			3.42
				
R posterior cingulate cortex	8 −40 26	0.042	323	4.31
R posterior cingulate cortex	2 −28 22			3.38
L thalamus	−6 −18 18			3.28
				
L lingual gyrus	−28 −56 10	0.017	451	4.25
L calcarine gyrus	−24 −68 10			3.71
L calcarine gyrus	−22 −38 22			3.67
				
R precuneus	32 −48 10	0.017	501	4.22
R precuneus	18 −42 4			4.08
R hippocampus	26 −34 0			3.90
				
R precuneus	18 −56 22	0.030	372	4.18
R precuneus	20 −54 34			3.58
				
R parahippocampal gyrus	34 −20 −26	0.248	165	4.00
R fusiform gyrus	26 -28 −24			3.95
R fusiform gyrus	38 -36 −22			2.91
				
R nucleus accumbens	14 8 −10	0.183	193	3.88
R ventral striatum	6 0 −12			3.69
R amygdala	14 −2 −8			3.47
				
L middle temporal gyrus	−52 4 −18	0.515	116	3.66
L middle temporal gyrus	−56 2 −28			3.31
				
*25>5-Point feedback*				
R ACC	2 34 14	0.872	118	3.92

Abbreviations: ACC, anterior cingulate cortex; EEG, electroencephalography; FDR, false discovery rate; fMRI, functional magnetic resonance imaging; L, left; R, right.

**Table 3:** EEG–fMRI coupling—theta-associated activations

*Anatomical area*	*Coordinates*	P*(FDR)*	*Size*	z*-score*
*Loss > gain*				
L superior parietal lobule	−18 −66 42	0.57	156	3.66
L superior parietal lobule	−24 −56 44			3.35
				
L ACC	−10 22 26	0.285	232	3.49
L ACC	−2 24 22			3.19
R ACC	8 24 24			3.11
				
R middle frontal gyrus	32 40 16	0.285	246	3.34
R middle frontal gyrus	26 40 30			3.33
R middle frontal gyrus	40 40 28			2.99
				
R superior temporal gyrus	46 −34 6	0.726	118	3.26
R superior temporal gyrus	54 −32 6			3.10
R middle temporal gyrus	44 −30 −2			2.93
				
L inferior parietal lobule	−42 −80 14	0.726	110	3.25
L inferior parietal lobule	−38 −68 14			3.21
				
*25>5*				
R middle temporal gyrus	52 −48 −4	0.207	277	4.04
R fusiform gyrus	34 −38 −18			3.59
R middle temporal gyrus	56 −38 −14			3.47

Abbreviations: ACC, anterior cingulate cortex; EEG, electroencephalography; FDR, false discovery rate; fMRI, functional magnetic resonance imaging; L, left; R, right.
